# Recent Updates on the Therapeutic Prospects of Reversion-Inducing Cysteine-Rich Protein with Kazal Motifs (RECK) in Liver Injuries

**DOI:** 10.3390/ijms242417407

**Published:** 2023-12-12

**Authors:** Giuseppina Palladini, Laura Giuseppina Di Pasqua, Anna Cleta Croce, Andrea Ferrigno, Mariapia Vairetti

**Affiliations:** 1 Department of Internal Medicine and Therapeutics, University of Pavia, Via Ferrata 9, 27100 Pavia, Italy; giuseppina.palladini@unipv.it (G.P.); lauragiuseppin.dipasqua01@universitadipavia.it (L.G.D.P.); mariapia.vairetti@unipv.it (M.V.); 2 Internal Medicine Fondazione IRCCS Policlinico San Matteo, Viale Camillo Golgi 19, 27100 Pavia, Italy; 3 Institute of Molecular Genetics, Italian National Research Council (CNR), Via Abbiategrasso 207, 27100 Pavia, Italy; annacleta.croce@igm.cnr.it; 4 Department of Biology & Biotechnology, University of Pavia, Via Ferrata 9, 27100 Pavia, Italy

**Keywords:** reversion-inducing cysteine-rich protein with Kazal motifs, RECK, MMPs, ADAMs, HCC, CCA, NASH, NAFLD, ischemia/reperfusion

## Abstract

The reversion-inducing cysteine-rich protein with Kazal motifs (RECK), a membrane-anchored glycoprotein, negatively regulates various membrane proteins involved in the tissue governing extracellular matrix (ECM) remodeling such as metalloproteases (MMPs) and the sheddases ADAM10 and ADAM17. The significance of the present review is to summarize the current understanding of the pathophysiological role of RECK, a newly discovered signaling pathway associated with different liver injuries. Specifically, this review analyzes published data on the downregulation of RECK expression in hepatic ischemia/reperfusion (I/R) injury, liver-related cancers, including hepatocellular carcinoma (HCC) and cholangiocarcinoma (CCA), as well as in the progression of nonalcoholic fatty liver disease (NAFLD) to non-alcoholic steatohepatitis (NASH). In addition, this review discusses the regulation of RECK by inducers, such as FXR agonists. The RECK protein has also been suggested as a potential diagnostic and prognostic marker for liver injury or as a biomarker with predictive value for drug treatment efficacy.

## 1. Introduction

**Figure 1:**
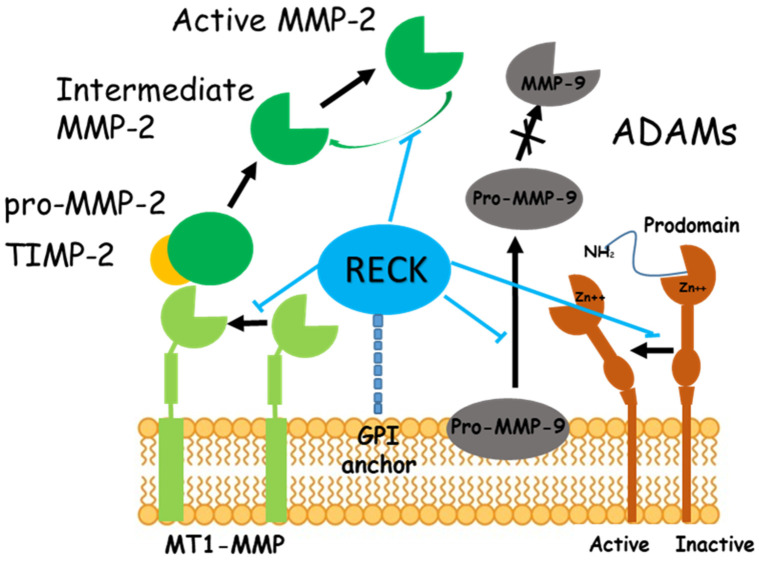
Effects of RECK on MMPs and ADAMs. RECK is associated with the membrane through GPI-anchoring. RECK inhibits both the release of pro-MMP-9 from the cells [2] and the enzyme activity of MT1-MMP and MMP-2, resulting in reduced production of active MMP-2 [1]. RECK also reduced ADAM10 and ADAM17 [10]. Protein containing a disintegrin and metalloprotease domain (ADAM); glycosylphosphatidylinositol (GPI); reversion-inducing cysteine-rich protein with Kazal motifs (RECK); tissue inhibitor of matrix metalloproteinase (TIMP)-2.

The reversion-inducing cysteine-rich protein with Kazal motifs (RECK) is a membrane-anchored glycoprotein that interacts with other membrane-associated proteins regulating their activity [1]. RECK was initially identified as a negative regulator of matrix metalloproteinase-9 (MMP-9) and it can sequester and prevent the activation of proMMP-9 due to the presence of protease inhibitor-like domains in its extracellular region [2]. In the last years, it has been shown that RECK regulates other members of the MMP family such as MMP-2, MMP-7, a matrilysin implicated in cardiac remodeling [3], MMP-17 or MT4-MMP that belongs to the membrane-type matrix metalloproteinases (MT-MMPs), anchored to the cell surface by a glycosylphosphatidylinositol (GPI) motif, whose expression is well documented in a variety of cancers [4]. Recently, it was shown that RECK could also interfere in the regulation of MMP-13, a stromelysin whose expression contributes to fibrosis [5]. RECK also promotes the pro-MT1-MMP processing to mature MT1-MMP, likewise known as MMP-14 [6]; interestingly, the association of RECK-MT1-MMP enhances the deposition of fibrillin and fibronectin [7]. RECK also plays an important role in extracellular matrix (ECM) remodeling by inhibiting the activation of MMP-2 and MMP-9, gelatinases dedicated to the cleavage of ECM components (Figure 1). The decreased activity of gelatinases, especially MMP-2, is related to the development of liver fibrosis, probably due to their proteolytic role, involved in maintaining the ECM balance [8].

RECK is not a direct inhibitor of the catalytic activity of MMPs, but it can regulate MMPs in vivo at different levels, e.g., through downregulation of the transcription, translation, or secretion of MMPs, or by preventing them from performing their extracellular peptidolytic function [9]. To date, it can be argued that the role of RECK is to slow down the remodeling mediated by MMPs.

RECK can also modulate the activity of the sheddases ADAM10 and ADAM17 [10] (Figure 1) which are involved in the shedding and activation of Notch, a functional link between RECK and CSCs cancer stem cells [11]. ADAM17, also known as TNF-alpha–converting enzyme (TACE), plays a pivotal role in inflammation [12], but has also emerged as a key regulator of several physiological and pathophysiological processes, such as cell proliferation, immune cell activation, cell death, inflammation, and cancer [12]. Since TNF-alpha is upregulated in the progression of nonalcoholic fatty liver disease (NAFLD) to non-alcoholic steatohepatitis (NASH), it is interesting to speculate that regulating the TNF-alpha release by targeting ADAM17 could serve as a therapeutic strategy to reduce liver inflammation.

It is known that ADAM10 functions as a “molecular scissor” playing a pivotal role in the inflammatory response by regulating proteolysis through the cleavage of various membrane protein substrates, including the activation of the pro-inflammatory Notch signaling cascade [13]. Notch activity, specifically in hepatocytes, correlates with NASH severity. As previously documented, the increased number of hepatocytes expressing major Notch signaling pathway products suggests that hepatocyte Notch activation may be a causative factor in NASH severity [14]. Since RECK modulates the Notch pathway via direct inhibitory regulation of both ADAM10 and ADAM17, it is possible to speculate that RECK modulation could have a potential therapeutic function in NASH.

ADAM10 and ADAM17 also play a crucial role in the regulation of the epidermal growth factor receptor (EGFR) signaling cascade [15,16]. It can therefore be hypothesized that the RECK-mediated inhibition of ADAMs could also prevent the release of membrane-anchored EGFR ligand, thus suppressing EGFR activation. Since EGFR is implicated in hepatocyte regeneration in HCC development, its RECK-mediated inhibition is very interesting in the context of NASH. Pharmacological inhibition of EGFR has been shown to reduce high-fat (HF) diet-induced liver injury in mouse models of NAFLD [17,18], suggesting that targeting EGFR signaling may hold promise as a potential therapeutic approach for NASH.

In liver fibrosis, the activation of HSCs produces TGF-β, IL-6, IL-1α, IL-1β, and IL-8 and other inflammatory mediators including chemokines [19]. How cytokines contribute to RECK-induced liver injury is still poorly studied. A recent study documented both a RECK decrease and a TGF-β increase in a diet-induced ob/ob mouse model of NASH [10]. In cardiac diseases, RECK has been shown to reduce several downstream effectors of Angiotensin II (Ang-II), such as Interleukin-6 (IL-6)/IL-6 receptor (IL-6R)/glycoprotein 130 (IL-6 signal transducer) signaling [20].

In addition, as also described above, in the last years some studies have confirmed that RECK levels are significantly downregulated in human malignancies and metastasis [6,21].

The present review summarizes the current understanding of the pathophysiological role of RECK, a relatively newly discovered signaling pathway associated with various liver injuries. In detail, published data on the downregulation of RECK expression and liver-related cancer, hepatocellular carcinoma (HCC) and cholangiocarcinoma (CCA), NAFLD progression to NASH and ischemia/reperfusion (I/R) injury have been reviewed, as well as the effects of RECK regulation by inducers.

## 2. Ischemia/Reperfusion (I/R) Damage and RECK

The term ischemia refers to an inadequate blood supply to tissues due to the obstruction of the arterial inflow. This condition is a common consequence of certain surgical procedures and pathologies. Several are the evidence supporting the concept that the reperfusion period, once blood circulation is re-established, induces and exacerbates tissue injury and necrosis with a complex series of events resulting in the recruitment of inflammatory cells, rearrangement of the ECM and induction of cell death, leading to organ dysfunction [22]. Despite years of intensive investigation, we are still far away from completely understanding the underlying mechanisms of ischemia/reperfusion (I/R) injury. MMPs, important regulators of many cellular activities including ECM remodeling, have been recognized for their central role in disease progression after I/R injury, as evidenced by numerous studies using MMP inhibitors or MMP-deficient mice [23]. Recently, the role of RECK, as a crucial regulator of MMPs in I/R damage, has been evaluated in the brain [24] and liver [25]. RECK is implicated in the protection of ECM/tissue integrity and the promotion of functional recovery in the brain after transient cerebral ischemia. Recent data provides new insights into RECK changes: a transitory decrease in RECK, associated with an increase in MMP activity, has been reported during reperfusion in hepatic I/R injury. These results suggest that RECK could be considered a triggering factor of organ dysfunction. In detail, a RECK downregulation was related to both MMP-2 and MMP-9 over-activation at 60 min reperfusion whereas no significant MMP activation was detectable at 120 min reperfusion when the upregulation of RECK expression was recorded [25].

The involvement of ERK1/2-, JNK-, and p38- MAPK signaling pathways has also been shown associated with the RECK downregulation under hypoxic conditions [26]. After 60 min of reperfusion, a RECK downregulation occurred in the liver, in association with a greater phosphorylation of MAPKs (ERK1/2, JNK1/2, and p38), which can be considered early events induced by I/R injury. While 120 min reperfusion caused an increase in RECK content and lower levels of phosphorylated MAPKs, with values similar to those observed in sham-operated animals. In liver I/R damage, the reperfusion was able to modulate the RECK expression accompanied by opposite changes in both MMP activity and MAPK activation [26].

It has been suggested that hepatic I/R injury may lead to damage also in remote organs, such as the kidneys [27]. Changes in tissue RECK and MMPs were evaluated in the kidney cortex and medulla after hepatic I/R; although, RECK levels showed no significant changes, an increase in MMP-9-dimer activity in the kidney cortex and medulla was found [28].

The modulation of RECK expression after I/R injury could be considered an innovative therapy in the reduction of tissue damage favoring a functional recovery.

**Figure 2:**
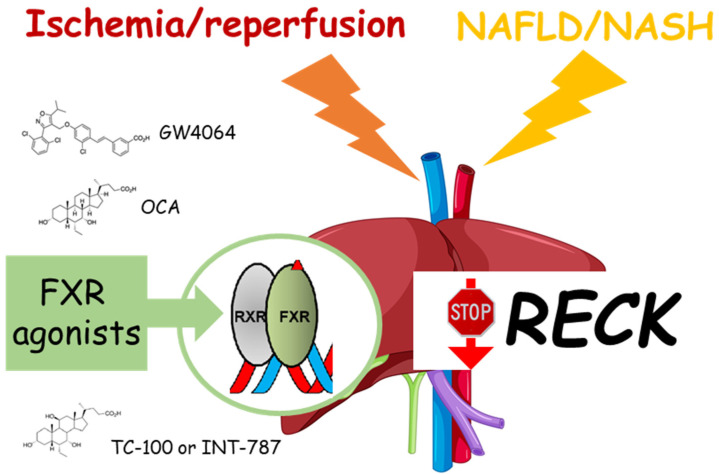
Effects of FXR agonists on RECK after ischemia/reperfusion damage or NAFLD/NASH. The administration of FXR agonists, such as GW4064, OCA, or TC-100 (INT-787), markedly counteracts the downregulation of RECK. Farnesoid X receptor (FXR); obeticholic acid (OCA).

Of note, RECK was identified as a novel target gene of farnesoid X receptor (FXR) agonists [29]. FXR belongs to the ligand-activated nuclear receptor superfamily and functions as a metabolic regulator and cell protector [30]. Recently, it has been demonstrated that the bile acid-derivative obeticholic acid (OCA), an FXR agonist, reduces the activation of MMP-2 and MMP-9 during hepatic I/R damage, via a timely recovery of the RECK protein [31] (Figure 2). Moreover, while no significant differences in RECK were observed, the administration of OCA in the kidney demonstrated beneficial effects by reducing TNF-alpha-mediated expression of MMPs after liver I/R [28].

## 3. NAFLD and RECK

NAFLD is a progressive disease whose onset and development depend on complex factors related to dysmetabolic diseases. In 2020, the name changed from NAFLD to metabolic-associated fatty liver disease (MAFLD) to refer to fatty liver disease related to systemic metabolic dysregulation. The superiority of the use of MAFLD terminology over NAFLD terminology is due to the inclusion of hepatic and extrahepatic mortality risk, disease associations, and the identification of high-risk individuals [32]. The peculiarity of MAFLD is due to the consideration of both liver-related events and extrahepatic events, principally cardiovascular and cancers [33]. Moreover, when compared to NAFLD, MAFLD is better recognized in patients with advanced liver fibrosis [33]. The major changes are due to the inclusion of individuals with significant alcohol intake or chronic viral hepatitis in the MAFLD criteria who have been excluded in the NAFLD criteria, and the exclusion of individuals with fatty liver without metabolic abnormality who have been included in the NAFLD criteria [34]. Recently, both MAFLD and NAFLD have been associated with an increased risk of HCC for individuals without other chronic liver diseases [34]. NAFLD/MAFLD progression to NASH/metabolic-associated steatohepatitis (NASH/MASH) is due to intrahepatic accumulation of toxic lipids involved in inflammation and oxidative stress [33].

During liver fibrosis development, there is a shift in the intrahepatic balance where ECM accumulation exceeds its degradation, resulting in excessive collagen deposition. Although the role of RECK in myocardial fibrosis has been thoroughly studied [20], its role in the development of hepatic fibrosis is still poorly understood. Previous studies have demonstrated downregulation of RECK expression in the livers of mice with Western diet (WD)-induced NASH. RECK mRNA and protein expression were significantly reduced [35]. The downregulation of RECK expression was associated with hepatic steatosis and fibrosis, elevated expression of genes involved in hepatic fibrogenesis (Tgfb1 and Col1α1), and MMPs [35]. Furthermore, in mice with hepatic knockdown of RECK, an increase in NASH susceptibility was detected, confirming the previous results. RECK knockdown exacerbated fibrosis and inflammation as well as the expression of MMPs [36].

A decrease in RECK expression was also confirmed in the livers of mouse and rat models of NASH induced by a methionine and choline-deficient (MCD) diet [29,37]. In particular, the time-dependent decrease in RECK levels was associated with an increase in MMPs during the progression from steatosis to steatohepatitis [37]. Recently, a downregulation of liver RECK mRNA and protein was also observed in an HF diet-induced ob/ob mouse model of NASH. These events were associated with an increase in MMP-2 and MMP-9 mRNA and activity, as well as in sheddases ADAM10 and ADAM17 [10].

Using an in vitro cell culture model employing isolated mouse primary hepatocytes, Dashek et al. (2022) tested whether the induction of RECK expression inhibited proinflammatory amphiregulin and EGFR signaling, the progression of NASH, and the development of HCC. RECK overexpression inhibited TNF-alpha-induced ADAM10/17 activity and downstreamed amphiregulin and EGFR signaling, under pro-inflammatory stimuli. Because increased EGFR activity contributes to the progression of NASH to HCC in preclinical models, these results indicate that inducing RECK has the potential to inhibit hepatocellular inflammation in the setting of NASH and HCC [38].

In the liver, RECK acts as a transcriptional target of FXR that plays a key role in bile acid, cholesterol, lipid, and glucose metabolism [29]. Therefore, FXR is a potential drug target for several metabolic disorders, especially those related to metabolic syndrome. Peng et al. demonstrated that the FXR agonist GW4064 can attenuate hepatic fibrosis and inflammation in MCD diet-fed mice through the FXR-RECK-MMP-9 cascade [29]. Recently, the administration of FXR agonists, the previously studied OCA, and the novel TC-100 (INT-787), markedly increased RECK mRNA expression and upregulated RECK protein in an HF diet-induced ob/ob mouse model of NASH [10] (Figure 2). The first evidence of FXR agonists reducing both MMP-2 and MMP-9 mRNA and activity, as well as inhibiting ADAM10 and ADAM17, was presented in this study. In detail, this study also showed that TC-100 had a stronger positive effect than OCA, by regulating both inflammatory and fibrogenic processes through RECK. This resulted in less lipid and collagen accumulation and better mitochondrial function to produce ATP. [10]. These results highlight the emerging role of RECK in regulating fibrogenic and inflammatory processes.

## 4. Cholangiocarcinoma (CCA) and RECK

Cholangiocarcinoma (CCA), also called bile-duct cancer, is a highly complex cancer characterized by the malignant growth of the epithelial lining in the bile ducts, which spans the entire biliary tree and is accountable for disease progression [39]. RECK gene expression is downregulated in many solid tumors and this downregulation is associated with poor prognosis [40]. In particular, RECK has been implicated in the attenuation of tumor metastasis by negatively regulating MMP levels and this role of RECK was also evaluated in CCA. In detail, CCA is recognized as a highly metastatic tumor, possibly due to its ability to secrete MMP-2 and MMP-9 [41]. Namwat et al. also reported the expression of RECK and MMPs in hamster and human CCA specimens as well as the functional analysis of RECK in RECK small interfering (si) RNA knockdown CCA cell lines [40]. Using hamster tissues, RECK was highly expressed in hyperplastic biliary duct epithelia, lowly expressed in precancerous lesions, and not expressed in CCA. A similar trend occurred in human specimens: high RECK expression was documented in normal biliary cells, whereas low levels of expression were found in intrahepatic CCA [40]. Furthermore, the silencing of RECK in human M139 CCA cells induced an increase in the secretion of both MMP-2 and MMP-9, resulting in an enhanced cell invasive ability [40].

Similar results were documented in patients showing significantly lower RECK expression in hilar CCA tissues than in normal bile duct tissues, in association with higher invasion levels of the liver and surrounding organs. The opposite trend occurred for MMP-9 expression which is highly expressed in hilar CCA tissues [42].

The effect of nonsteroidal anti-inflammatory drugs (NSAIDs) on RECK levels has been evaluated in human CCA cell lines in a study that documented the induction of RECK by NSAIDs in lung cancer cells [43]. Aspirin was able to upregulate RECK levels, inhibit MMP-2/MMP-9 activity, and reduce the number of invasive CCA cells through the inhibition of the Ras signaling pathway by reducing the phosphorylation of Akt/Erk/c-Jun in CCA cells [40]. Of note, it has been reported that the RECK changes in malignant cells involve the activation of oncogenic Ras signaling, including the Raf/Mek/Erk and Mekk/Sek/Jnk pathways [14,44].

Previous studies support the potential role of microRNA (miR), noncoding RNA molecules that post-transcriptionally regulate gene expression, as therapeutic targets. Loayza-Puch et al. reported that RECK is a target of at least three groups of miRNAs (miR-15b/16, miR-21, and miR-372/373), and the hypoxia- and RAS-signaling pathways downregulate RECK through miRs thereby promoting malignant cell behavior [45].

The role of onco-miR-21 has been evaluated in the genesis of Opisthorchiasis-associated CCA showing that miR-21 was upregulated during cholangiocarcinogenesis not only in the hamster model but also in human CCA samples. An inverse correlation between miR-21 levels versus RECK mRNA has been reported in human CCA. The knockdown of miR-21 in KKU100 CCA cells showed an increase in RECK mRNA level as well as a suppression of CCA cell migration [46]. The potential role of miR-21 in CCA growth and metastasis has been further evaluated by Huang et al. [47]. In patients with lymph node metastasis or perineural invasion, a significantly higher expression of miR-21 was found. A decrease in CCA cell line invasion and metastasis ability was documented after miR-21 knockdown. In addition, miR-21 potentially inhibited RECK expression in CCA cells [47].

Tumor cell-derived extracellular vesicles (EVs) may contribute to the development of malignancies by delivering molecular cargo miRs. Recently, Fu et al. reported that tumor cell-derived EVs can deliver miR-210 to CCA cells, where miR-210 can target and inhibit the expression of RECK thus promoting the growth, metastasis, and chemoresistance in CCA [48].

Hepatocyte nuclear factor 6 (HNF6) is a liver-enriched transcription factor and is highly expressed in mature bile duct epithelial cells. HNF6 has been found to inhibit the migration and invasion of CCA cells by regulating RECK and MMPs through miR-122 and its overexpression may represent a mechanism-based therapy for CCA [49].

## 5. Hepatocellular Carcinoma (HCC) and RECK

Recently, in a large sample of asymptomatic adults, both MAFLD and NAFLD are associated with an increased risk of hepatocellular carcinoma (HCC) for individuals without other chronic liver diseases [34].

In many types of cancer, the downregulation of RECK gene expression related to the expression and the activity of MMPs, such as MMP-2 and MMP-9, has been demonstrated [40]. These events were positively associated with tumor progression, invasiveness, metastasis, poor prognosis, and shorter patient survival time [50].

The involvement of RECK in HCC has been documented with opposite evidence. Furumoto’s study reported that HCC tissues expressed higher RECK mRNA than the noncancerous liver tissues [51], although it also described that cases with high expression of RECK mRNA exhibited better survival and less invasive tumors. Indeed, Zhang et al. demonstrated that RECK promoter hypermethylation was frequently observed in HCC and is associated with loss of mRNA expression; in addition, the decreased RECK mRNA is significantly correlated with worse survival in HCC [52]. Recently, in a large cohort of HCC patients, although no significant differences between HCC and normal liver regarding RECK mRNA levels were found, improved survival was reported in HCC patients with high RECK mRNA [53]. In addition, the authors documented a negative correlation of RECK with angiogenesis and a positive correlation with the expression of immune checkpoint molecules such as programmed death ligand 1 (PD-L1). Because PD-L1 is significantly high in RECK-positive HCC, this event might reflect an immunogenic status in HCC with the recruitment of more tumor-infiltrating lymphocytes [53]. Because angiogenesis and immunosuppression have been reported as connected processes that can occur in parallel, the functional inhibition of MMPs by RECK can suppress tumor growth not only through the reduction of angiogenesis but also through immune-related mechanisms [53].

Controversial results are also reported on the possible association between RECK gene single nucleotide polymorphisms (SNPs) and the development of HCC. In 2022, a study demonstrated no significant association between RECK and SNPs with the development and characteristics of hepatitis B-related HCC in Egyptian patients [54]. The analysis of RECK gene rs10814325 SNP revealed that it could not be considered a risk factor for HCC development in the presence of HCV infection, but may be related to the disease progression and metastasis [55]. Although a larger cohort of patients is required for more conclusive results, some studies suggested that RECK rs10814325 polymorphisms may affect the risk of developing HCC in an HBV-free Chinese population [56]. The same occurred in a case–control study performed in the Taiwanese population to evaluate the impact of polymorphisms in the RECK gene with HCC risk [57]. Also, Nasser et al. reported that the RECK gene rs10814325 is associated with higher HCC susceptibility [58]. In 2012, a study described that after adjusting for other co-variants, the individuals carrying RECK promoter rs10814325 inheriting at least one C allele had a 1.85-fold risk of developing HCC compared to the TT wild type carriers. The HCC patients who carried rs11788747 with at least one G allele had a higher distant metastasis risk than wild type probands [57]. RECK rs11788747 A/G and G/G genotypes frequencies were also evaluated and they were significantly higher in HCC patients compared to the healthy controls in the Egyptian population [59]. Another study investigated the role of tumor suppressor genes RASSF1A Ala133Ser and RECK rs11788747polymorphism in HCC. In the Egyptian population, a significant association between RASSF1AAla133Ser polymorphism and risk of HCC was found where A1a/Ser, Ser/Ser genotypes and Ser allele are risk factors for HCC development [60]. Similar results have been obtained in Turkish patients in which RASSF1AAla133Ser polymorphism was associated with increased susceptibility to HCC [61].

Of note, miRs, often localized to chromosomal fragile sites, are associated with cancer [62]. It has been widely demonstrated that miR-21 can function as an oncogene, increasing tumor cell migration and invasion by directly targeting phosphatase and tensin homolog deleted from chromosome 10 (PTEN), RECK, and programmed cell death 4 (PDCD4) [63,64]. In HCC cells, miR-21 simultaneously regulates multiple programs that enhance tumor invasiveness by targeting PTEN, RECK, and PDCD4 [63]. The same was determined by Zhou et al.: miR-21 overexpression drastically inhibited PTEN, RECK, or PDCD4 protein expression, while silencing miR-21 an increase in PTEN, RECK, or PDCD4 protein was found [65]. These authors observed that HCC cell migration and invasiveness weakened by anti-miR-21 was ‘rescued’ by knockdown of PTEN, PDCD4, or RECK. The biological effects of miR-21 on HCC cell invasion are probably due to the simultaneous repression of migration suppressive proteins such as PTEN, RECK, or PDCD4 [65].

Because liver inflammation plays a critical role in HCC etiology, the dysregulated miRs involved in inflammation, such as miR-21, and the damage-associated molecular patterns (DAMP), such as high-mobility group box 1 (HMGB1), have been evaluated. The release of HMGB1, during liver inflammation, has been found to increase miR-21 expression associated with enhanced activity of MMPs through RECK and the generation of a favorable environment for HCC growth [66]. Activation of STAT3 signaling, crucial in development and carcinogenesis, has been also shown to upregulate the expression levels of miRs, including miR-21 [67]. Interleukin-6-dependent survival of multiple myeloma cells involves the STAT3-mediated induction of miR-21, through a highly conserved enhancer. Of note, miR-21 is overexpressed in a variety of malignancies and linked to cell metastasis through its targets such as RECK. Previous findings indicated that STAT3 and phospho-STAT3 acted as an upstream regulators of miR-21 in human HCC cell lines, as they regulated cell metastasis-related capacities through targets of miR-21 and RECK [68].

Other miRs were evaluated in HCC: RECK was identified as the direct and functional target of miR-135b. The heat shock transcription factor 1 (HSF1) directly activated miR-135b expression, consequently enhancing HCC cell motility and invasiveness. The HSF1/miR-135b/RECK axis was thus found as an additional insight into the mechanisms of HCC metastasis [62].

Of note, long noncoding RNAs (lncRNAs), defined by a length greater than 200 nucleotides and limited protein-coding potential, also exhibit important regulatory roles in cellular functions and carcinogenesis [69]. Increasing evidence confirmed that dysregulation of lncRNAs is associated with multiple human cancers [70]. Zhang et al. reported that the level of LINC01419, a lncRNAs, is elevated in HCC tissues and associated with a malignant phenotype [71]. In vitro experiments demonstrated that LINC01419 silencing inhibited proliferation and migration in HCC cells in association with a significant increase in RECK [71]. Of note, lncRNA growth arrest-specific 5 (GAS5), a tumor suppressor in numerous kinds of human cancers, is a target of miR-135b. Furthermore, GAS5 positively regulates the expression of RECK, also a target of miR-135b, which further inhibits MMP-2 expression and contributes to invasion repression [72].

A recent study reported that mortalin, an oncogene, played a role in the migration and invasion of HCC cells through the regulation of RECK/STAT3 pathway; salvianolic acid B, a caffeic acid phenethyl ester analog, was able to increase the degradation of mortalin through ubiquitination, thereby upregulating RECK with a downregulation of MMP-2 and MMP-9, inhibiting STAT3, and inhibiting the migration and invasion of HCC cells [73].

Transmembrane protease serine 4 (TMPRSS4) is upregulated in a broad spectrum of cancers and its biological effects on HCC have been evaluated. TMPRSS4 overexpression suppressed RECK expression together with increased tumor angiogenesis, through activating the ERK1/2 pathway as well as promoted metastasis of HCC cells in vivo [74].

The chemopreventive effects of Polyphenon-B on markers of invasion and angiogenesis have been evaluated during dimethylaminoazobenzene (DAB)-induced hepatocarcinogenesis. The administration of Polyphenon-B significantly reduced the incidence of DAB-induced hepatomas as evidenced by the modulation of MMP-2, MMP-9, tissue inhibitor of matrix metalloproteinase (TIMP)-2, and RECK as well as angiogenesis (Table 1) [75].

## 6. Viral Hepatitis and RECK

In addition to metabolic disorders, other chronic liver injury such as viral hepatitis, is a worldwide health concern [76]. It is well known that Kupffer cells are activated by viral-derived pathogen-associated molecular patterns through toll-like receptor (TLR)-3 and TLR9 in chronic viral hepatitis [77]. Recently data demonstrated that in Hepatitis C virus (HCV) infection, the TLR signaling pathway causes various effector cells to produce inflammatory cytokines such as TNF-α, IL-1β, IL-6, IL-8, IL-12, and IL-18, involved in the activation of immune functions [78]. Interestingly, regulation of TLR mRNA has been shown to depend on one or more components of the MAPK pathway [79] and higher phosphorylation levels of hepatic MAPKs (ERK1/2, JNK1/2, and p38) have been demonstrated associated with RECK downregulation in liver I/R injury [25]. Previous insights into the molecular basis of viral hepatitis revealed that HBV, HCV, and HEV modulate the MAPK signaling pathway [80].

Because the involvement of ECM remodeling with increased expression and activation of MMPs is reported in other viral infections such as after coxsackievirus-induced myocarditis [81] as well as the dysregulations of RECK and MMP9 in Epstein-Barr virus infection in nasopharyngeal carcinoma [82], it is plausible to suppose that RECK may be involved in viral hepatitis via MAPK signaling.

A recent study evaluated the epigenetic regulatory mechanism of the RECK gene in HCV-related HCC patients. The authors reported that a RECK gene promoter hypermethylation is associated with HCV genotype-4-related HCC, thus hypothesizing a link between the methylation state of the RECK gene and hepatocarcinogenesis in chronically HCV-infected patients [83].

## 7. Future Prospective

The pathogenesis of several liver injuries is related to a downregulation of RECK. The overexpression or induction of RECK may have therapeutic potential in hepatic fibrogenesis, inflammation, and damage linked to NAFLD/NASH as well as to hepatic I/R injury. Targeting RECK, using for example FXR agonists, thus represents a promising therapeutic strategy in NAFLD or I/R disease that warrants further preclinical and clinical investigations.

Similar results obtained in HCC and CCA patients were found in other cancers such as patients with gastric cancer [84], colorectal cancer [85], and pancreatic cancer [21]: a significant negative correlation was found between RECK and MMP-2/MMP-9 expression. The downregulation of RECK expression might be one of the molecular mechanisms of CCA and HCC metastasis: when RECK is absent or diminished, MMPs are highly active, facilitating tumor growth and invasion. Indeed, forced expression of RECK in tumor cells results in a decreased incidence of malignancies in animal models. Moreover, because the reduced RECK expression is a key event for CCA and HCC progression, it may be regarded as a potential prognostic marker of patient survival time. Although large-scale prospective studies are needed, RECK may act as an indicator for the evaluation of efficacy using both anti-angiogenic therapy and immune checkpoint inhibitor therapy. Furthermore, the upregulation of RECK may be a promising approach for treating malignant tumors, also considering the RECK gene as a promising target for the prevention and treatment of hepatic cancer invasion and metastasis.

In addition, the evaluation of the contribution of RECK in viral hepatitis deserves to be studied.

Although RECK has also been reported to exert anti-inflammatory effects, further studies on the role of cytokines and RECK should be conducted; furthermore, the association between chemokines and RECK expression in the liver has been poorly studied.

The mechanisms and consequences of RECK downregulation in various liver injuries remain unclear. Therefore, experimental studies are needed to elucidate how this event occurs and what signaling pathways are affected by the low RECK levels in damaged liver tissue. This could lead to a better understanding of complex and multifaceted signaling pathways, such as those involved in metastatic processes as well as in viral hepatitis.

## 8. Conclusions

This review not only sheds light on the complex interplay between RECK, MMPs, and ADAMs but also highlights the potential of these proteins/enzymes as therapeutic targets. By integrating these perspectives, it will be possible to take steps forward in the management of different liver injuries, offering the prospect of slowing or controlling hepatic disease progression.

## Figures and Tables

**Table 1:** Potential inducers of RECK obtained from experimental studies.

Compound	Mechanism	Disease
FXR agonist	Decreased MMPs and ADAMs	I/R, NAFLD/NASH
NSAIDs (aspirin)	Inhibited MMP activity	CCA
Salvianolic acid B	Inhibition STAT3 pathway; downregulation of MMP-2 and MMP-9	HCC
Polyphenon-B	Decrease MMPs, TIMPs, and angiogenesis	HCC

## Data Availability

Not applicable.
